# Machine learning-based clustering in cervical spondylotic myelopathy patients to identify heterogeneous clinical characteristics

**DOI:** 10.3389/fsurg.2022.935656

**Published:** 2022-07-25

**Authors:** Chenxing Zhou, ShengSheng Huang, Tuo Liang, Jie Jiang, Jiarui Chen, Tianyou Chen, Liyi Chen, Xuhua Sun, Jichong Zhu, Shaofeng Wu, Zhen Ye, Hao Guo, Wenkang Chen, Chong Liu, Xinli Zhan

**Affiliations:** Department of Spine and Osteopathy Ward, The First Affiliated Hospital of Guangxi Medical University, Nanning, Guangxi, China

**Keywords:** machine learning, anterior cervical discectomy and fusion, anterior cervical corpectomy and fusion, cervical spondylotic myelopathy, cluster analysis

## Abstract

**Background:**

Anterior cervical decompression and fusion can effectively treat cervical spondylotic myelopathy (CSM). Accurately classifying patients with CSM who have undergone anterior cervical decompression and fusion is the premise of precision medicine. In this study, we used machine learning algorithms to classify patients and compare the postoperative efficacy of each classification.

**Methods:**

A total of 616 patients with cervical spondylotic myelopathy who underwent anterior cervical decompression and fusion were enrolled. Unsupervised machine learning algorithms (UMLAs) were used to cluster subjects according to similar clinical characteristics. Then, the results of clustering were visualized. The surgical outcomes were used to verify the accuracy of machine learning clustering.

**Results:**

We identified two clusters in these patients who had significantly different baseline clinical characteristics, preoperative complications, the severity of neurological symptoms, and the range of decompression required for surgery. UMLA divided the CSM patients into two clusters according to the severity of their illness. The repose to surgical treatment between the clusters was significantly different.

**Conclusions:**

Our results showed that UMLA could be used to rationally classify a heterogeneous cohort of CSM patients effectively, and thus, it might be used as the basis for a data-driven platform for identifying the cluster of patients who can respond to a particular treatment method.

## Introduction

The neurological symptoms of cervical spondylotic myelopathy (CSM) are mainly caused by spinal cord compression and can be relieved by surgical decompression (1,2). CSM was included in the umbrella term degenerative cervical myelopathy (DCM). DCM is a degenerative disease, and the process of disease progression is progressive and irreversible. Progressive compression of the cervical spinal cord by degeneration and spinal canal stenosis is the pathogenesis of DCM (3). Preventing symptom progression is one of the aims of surgery (4). After decompression of the cervical spinal cord, the pressure reduces, and some functions improve. Patients with more severe symptoms often show greater improvement in function (5,6). Anterior cervical decompression and fusion, including anterior cervical discectomy and fusion (ACDF) and anterior cervical corpectomy and fusion (ACCF), can effectively relieve cervical spinal cord compression and improve neurological symptoms (7). With the advancement in the medical field, precision medicine has greatly enhanced the effectiveness of medication and reduced the incidence of patient complications. Accurately identifying the problem and classifying the patient has become very important (8). With the development of artificial intelligence, machine learning has been applied to disease diagnosis, classification, and treatment, such as heart failure and child-specific dermatitis (8,9).

Supervised machine learning utilizes iterative algorithms and learns from a large and accurately labeled training dataset (10). Although it can accurately diagnose diseases, it is generally unable to infer “diagnostic reasoning” used in these algorithms. Unsupervised machine learning algorithms (UMLAs) cluster (or group) patients based on their characteristics rather than identifying the “truth” of diagnosis or prognosis (11). By clustering patients, it is possible to analyze the characteristics of similarly clustered individuals and determine the outcome of therapy.

Machine learning algorithms are widely used in clinical practice (12,13) but are seldom used to evaluate the efficacy of cervical spine surgery. It is important to effectively classify patients undergoing cervical surgery and identify their potential risks (14). Unsupervised machine learning algorithm (UMLA) can be used to cluster patients based on their disease characteristics and accurately and rationally classify a heterogeneous cohort effectively (15). We rationally classify a heterogeneous cohort of patients by UMLA and form a basis for a data-driven platform that can identify the subgroup of patients who might respond to a particular treatment. Eventually, the management of CSM patients including treatment decision making can be improved. In this study, we collected the clinical data of patients with CSM who underwent anterior cervical decompression and fusion. UMLA was performed to classify patients into two clusters based on the characteristics of the perioperative clinical data of the patients. Finally, we compared the differences in the clinical characteristics, the efficacy of the surgery, and postoperative complications between the clusters and verified the accuracy of the clustering performed.

## Materials and methods

### Patients

This study was authorized by the Ethics Committee of The First Affiliated Hospital of Guangxi Medical University. Informed consent forms were signed by the volunteers who participated in this study.

We collected the clinical data of the CSM patients who were hospitalized and underwent anterior cervical decompression and fusion (ACDF and ACCF) from June 2012 to June 2021 in the Department of Spinal Osteopathology, The First Affiliated Hospital of Guangxi Medical University. In total, 616 patients were enrolled in this study. We collected data on 31 perioperative variables, including 13 preoperative variables and 18 operative and postoperative variables. The 13 preoperative variables were gender, age, body mass index (BMI), the American Spinal Cord Injury Association (ASIA) score, the visual analog scale (VAS), stability of the cervical spine, segments of the spinal operation, and history of diabetes, osteoporosis, hypertension (HP), cardiovascular heart disease (CHD), cerebrovascular disease (CVD), and hepatic and renal function disorder (HRFD). The 18 operative and postoperative variables included respiratory failure, peptic ulcer postoperation, dysphagia, pneumonia, hoarseness, mental disorder, axial pane, leakage of cerebrospinal fluid, esophagostoma, wound hematomas, sense of girdle, wound infection, JOA improvement rate >25%, blood transfusion, operation time (OT), bleeding volume (BV), postoperative drainage volume (PDV), and the length of hospital stay (LOS). The inclusion criteria were as follows: (I) The inpatient was diagnosed with CSM. (II) The inpatient underwent anterior cervical decompression and fusion, including anterior cervical discectomy and fusion (ACDF) and anterior cervical corpectomy and fusion (ACDF). (III) There were symptoms, signs, and imaging manifestations of cervical spinal cord compression in hospitalized patients. (IV) All patients who completed the preoperative examination. (V) The anterior cervical decompression and fusion operation was completed. The exclusion criteria were as follows: (I) Inpatients with tumor, tuberculosis, and space-occupying lesions in the cervical medulla were excluded. (II) Inpatients with traumatic cervical spinal cord injury. (III) Inpatients with cervical spondylosis who received other approaches, such as a posterior approach or a combined (anterior and posterior) approach. (IV) Inpatients without complete perioperative clinical data.

### Normalization of data and unsupervised machine learning

We used R software 4.0.3 to perform unsupervised machine learning. The data of anterior cervical decompression and fusion patients were normalized using the Scale function in the “factoextra” package (16). The Fpc package was used for determining the optimal clustering number (*K* value) by the Silhouette coefficient (SC) (17,18). Then, we used the K-means cluster algorithm to cluster the patients (13,18,19). The result of the K-means cluster was visualized using a clustergram and a radargram.

### Statistical analysis

SPSS V.22.0 was used for performing statistical analyses. The clinical measurement data of the patients were represented as the median (P25, P75) and the mean (SD). We performed a chi-squared test, Mann–Whitney *U*-test, and Student's *t*-test to compare the differences. The differences were considered to be statistically significant at *p* < 0.05.

## Results

### Result of unsupervised machine learning algorithms

The K-means clustering algorithm was used to identify and classify the characteristic clinical data of the patients. It is a common unsupervised algorithm in machine learning. It can categorize the data of unknown labels into different groups based on their characteristics. Each group of data is also called a “cluster,” and the center point of each cluster is called a “centroid.” After normalizing the clinical data using the Scale function, we used the K-means clustering algorithm. The basic process is as follows: (I) The K initial centroids (that may not be sample points) are randomly selected, the nearest centroid for each sample point is found, and the sample points and centroids are grouped into the same cluster, thus generating K clusters. (II) When all sample points are divided, for each cluster, the new centroid (the average coordinate value of all points in the same cluster) is recalculated. (III) Iterations are performed until the position of the centroid becomes constant. In this study, we determine the K value using the SC


$$\mathrm{SC}(i)=\frac{b(i)-a(i)}{\max\;\left\{a(i),b(i)\right\}}$$

**Figure 1:**
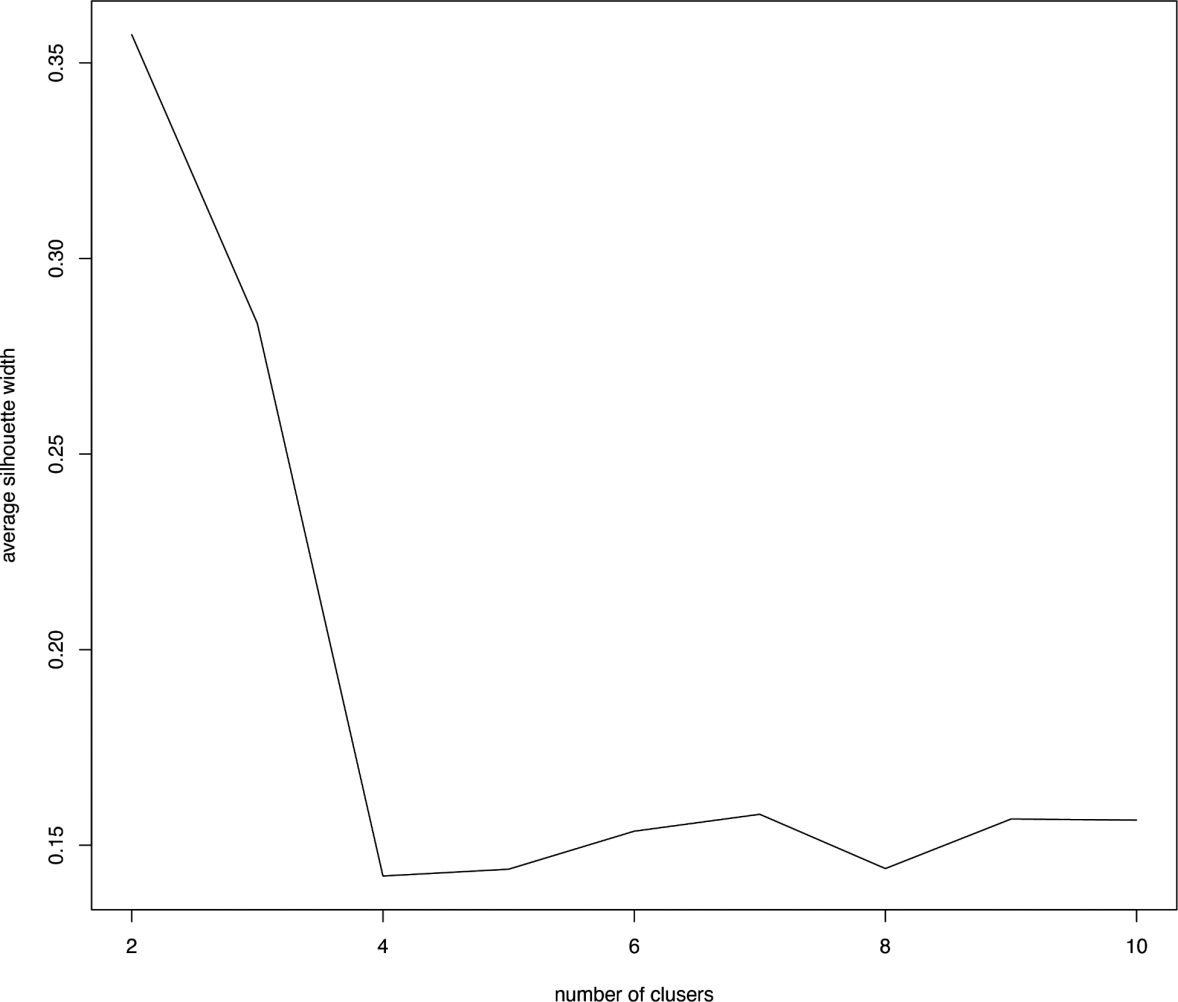
Optimal clustering number of the K-means clustering algorithm was determined by Silhouette coefficient (SC). The peak of the curve is the best value for the Silhouette coefficient (*Y*-axis); the best number of clusters was equal to 2 (*X*--axis).

**Figure 2:**
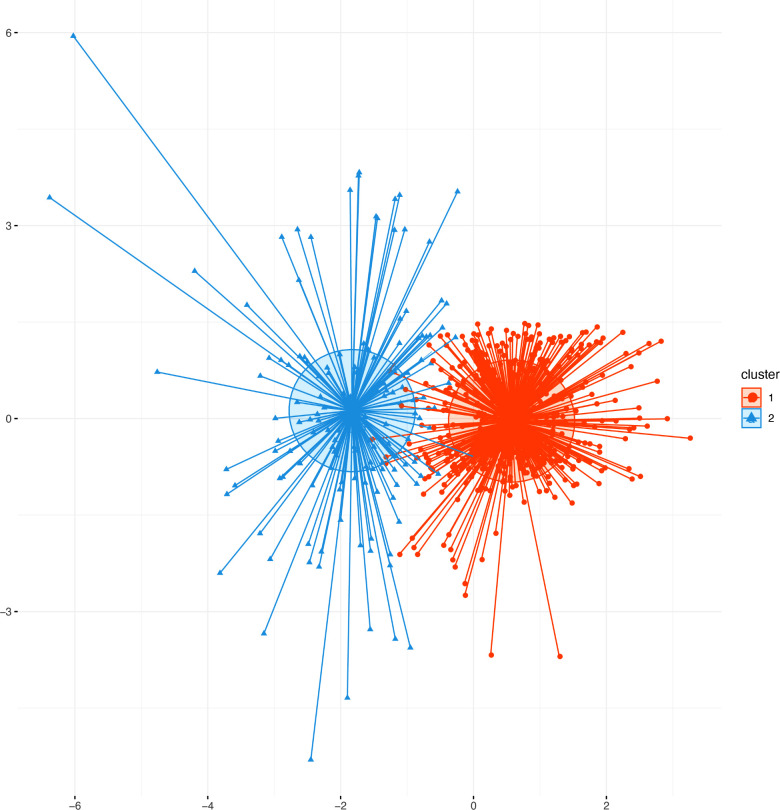
Scatter plots of patients’ clinical data. Scatter points on the graph represent each patient, and the K-means clustering algorithm divides patients into two clusters. The orange scatter represents cluster 1, and the blue scatter represents cluster 2.

**Figure 3:**
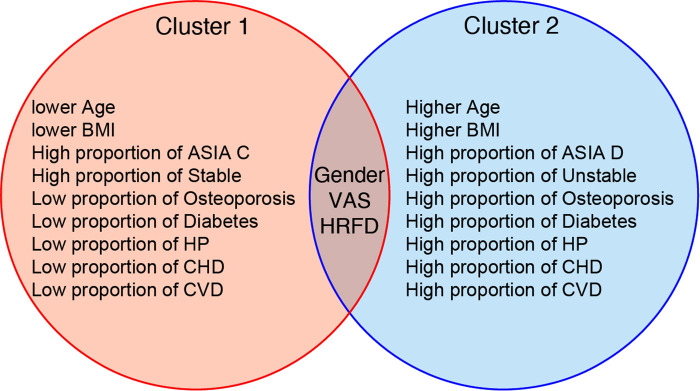
Typical clinical characteristics and features of two clusters. The blue circle represents cluster 1, more severe in condition, as opposed to the red circles. The Venn diagram summarizes the results of the unsupervised machine learning algorithm (UMLA). The results of UMLA are clinically explicable. BMI: body mass index; ASIA: American Spinal Cord Injury Association; HP: hypertension; CHD: cardiovascular heart disease; CVD: cerebrovascular disease; VAS: visual analog scale; HRFD: hepatic and renal function disorder.

In this formula, *a*(*i*) represents the average distance between the sample point and all other points in the same cluster and *b*(*i*) represents the average distance between the sample point and all points in the next nearest cluster (20). The K-means clustering algorithm pursues that, for each cluster, the difference within the cluster is small, while the difference between clusters is large, and the Silhouette coefficient is the key indicator to describe the difference inside and outside the cluster. According to the formula, the value of SC can range from −1 to 1. When SC is closer to 1, the clustering effect is better; when SC is closer to −1, the clustering effect worsens. The peak of the curve is the best value for the Silhouette coefficient (*Y*-axis) such that the best K value is equal to 2 (*X*-axis) (Figure 1). Two clusters are optimal for the K-means clustering algorithm. The clinical data were sufficiently clustered into cluster 1 and cluster 2 (Figure 2). The results of the K-means clustering algorithm for the clinical data are presented in Table 1. The heterogeneity and homogeneity of the clinical characteristics of the two clusters were determined using a Venn diagram (Figure 3).

**Table 1:** Baseline characteristics of the study patients by clusters.

	Overall (*n* = 616)	Cluster 1 (*n* = 469)	Cluster 2 (*n* = 147)	*p*-value
Gender				0.301
Male	362 (58.77%)	281 (59.91%)	81 (55.10%)	
Female	254 (41.23%)	188 (40.09%)	66 (44.90%)	
Age				<0.001
Mean ± SD	54.25 ± 10.06	52.03 ± 9.32	61.36 ± 9.01	
Medium [P25, P75]	54 [48,60]	52 [47,57]	60 [56,68]	
BMI				<0.001
Mean ± SD	23.21 ± 3.10	22.72 ± 2.89	24.77 ± 3.26	
Medium [P25, P75]	22.86 [21.37,24.93]	22.49 [20.96,24.27]	24.77 [23.30,26.62]	
ASIA				0.030
C	230 (33.34%)	164 (34.97%)	66 (44.90%)	
D	386 (62.66%)	305 (65.03%)	81 (55.1%)	
VAS				0.355
Mean ± SD	3.65 ± 2.02	3.69 ± 2.01	3.52 ± 2.05	
Medium [P25, P75]	3 [2,6]	3 [2,6]	3 [2,5.5]	
Stability				<0.001
Stable	550 (89.29%)	442 (94.24%)	108 (73.47%)	
Unstable	66 (10.71%)	27 (5.76%)	39 (26.53%)	
Segment				<0.001
1	75 (12.18%)	67 (14.29%)	8 (5.44%)	
2	407 (66.07%)	320 (68.23%)	87 (59.18%)	
3	120 (19.48%)	77 (16.42)	43 (29.25%)	
4	14 (2.27%)	5 (1.06%)	9 (6.13%)	
Osteoporosis	22 (3.57%)	0 (0%)	22 (14.97%)	<0.001
Diabetes	40 (6.49%)	15 (3.20%)	25 (17.01%)	<0.001
HP	104 (16.88%)	1 (0.21%)	103 (70.07%)	<0.001
CHD	3 (0.48%)	0(0%)	3 (2.04%)	0.002
HRFD	1 (0.16%)	0(0%)	1 (0.68%)	0.074
CVD	10 (1.62%)	3 (0.64%)	7 (4.76%)	<0.001

BMI: body mass index; ASIA: American Spinal Cord Injury Association; HP: hypertension; CHD: cardiovascular heart disease; CVD: cerebrovascular disease; VAS: visual analog scale; HRFD: hepatic and renal function disorder.

### Characteristics of the patients by K-means clustering

**Figure 4:**
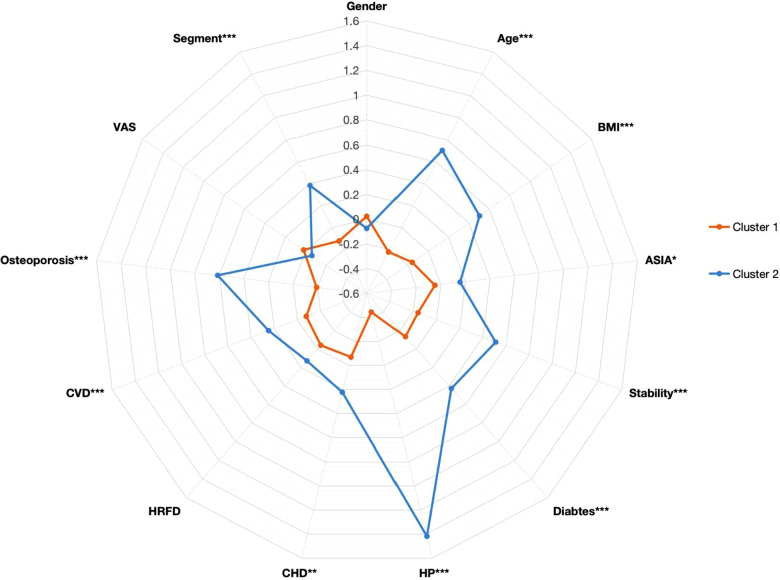
Radargram of 13 preoperative variables in cervical spondylotic myelopathy patients in two clusters. The K-means clustering algorithm normalized preoperative variables were compared between two clusters. Spoke lengths represent the average of each variable after the K-means clustering algorithm is normalized. Significance levels are presented with asterisks. BMI: body mass index; ASIA: American Spinal Cord Injury Association; HP: hypertension; CHD: cardiovascular heart disease; CVD: cerebrovascular disease; VAS: visual analog scale; HRFD: hepatic and renal function disorder. *p-value <0.05, **p-value <0.01, ***p-value <0.001.

The 13 preoperative variables of the two clusters based on the K-means clustering algorithm are presented in Table 1. These variables included gender, age, BMI, the ASIA score, the VAS, stability of the cervical spine, segments of the spinal operation, and history of diabetes, osteoporosis, HP, CHD, CVD, and hepatic and renal function disorder (HRFD). The age and BMI of cluster 1 were significantly lower than those of cluster 2 (age: cluster 1/cluster 2 = 52.03 ± 9.32/61.36 ± 9.01, *p* < 0.001; BMI: cluster 1/cluster 2 = 22.72 ± 2.89/24.77 ± 3.26, *p* < 0.001). The ASIA score, stability of the cervical spine, and segments of the spinal operation were significantly different between cluster 1 and cluster 2 [ASIA score-C grade: cluster 1/cluster 2 = 164 (34.97%)/66 (44.90%), *p* = 0.030; instability of the cervical spine: cluster 1/cluster 2 = 442 (94.24%)/108 (73.47%), *p* < 0.001]. History of diabetes, osteoporosis, HP, CHD, and CVD were significantly different between cluster 1 and cluster 2 [diabetes: cluster 1/cluster 2 = 15 (3.20%)/25 (17.01%), *p* < 0.001; HP: cluster 1/cluster 2 = 1 (0.21%)/103 (70.07%), *p* < 0.001; CHD: cluster 1/cluster 2 = 0 (0%)/3 (2.04%), *p* = 0.002; CVD: cluster 1/cluster 2 = 3 (0.64%)/7 (4.76%), *p* < 0.001]. The preoperative variables are represented by a radargram in Figure 4.

### Comparison of operative and postoperative variables among clusters

**Figure 5:**
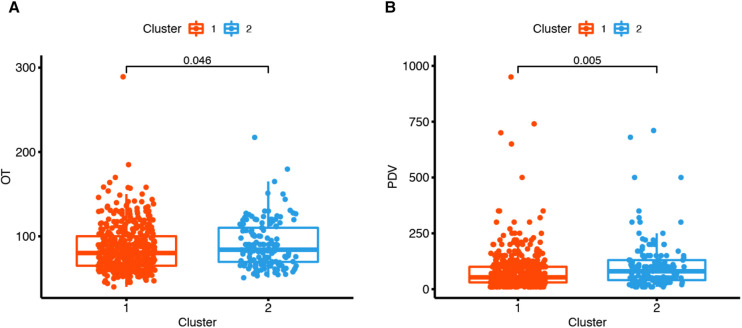
Two clustered boxplots of operative time and postoperative drainage volume. (**A**) The operation time (OT) of cluster 1 was 80[65, 100] min, and that of cluster 2 was 84[69, 110] min. The operation time of cluster 1 was significantly shorter than that of cluster 2 (*p* = 0.046). (**B**) The postoperative drainage volume (PDV) of cluster 1 was 53[30, 100] ml, and that of cluster 2 was 80[40, 130] ml. The postoperative drainage volume of cluster 1 was significantly less than that of cluster 2 (*p* = 0.005).

**Figure 6:**
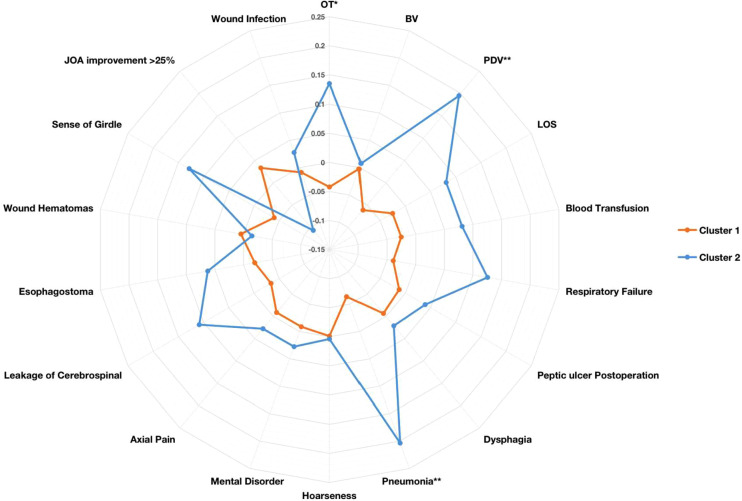
Radargram of 18 preoperative variables in cervical spondylotic myelopathy patients in two clusters. K-means clustering algorithm-normalized preoperative variables were compared between two clusters. Spoke lengths represent the average of each variable after the K-means clustering algorithm is normalized. Significance levels are presented with asterisks. OT: operation time; BV: bleeding volume; PDV: postoperative drainage volume; LOS: length of hospital stay. *p-value < 0.05, **p-value < 0.01, ***p-value < 0.001.

The differences in the 18 operative and postoperative variables between the clusters are presented in Tables 2 and 3. The incidence of postoperative pneumonia in cluster 2 was higher than that in cluster 1 (*p* < 0.05). The OT of cluster 1 was 80 [65, 100] min, and that of cluster 2 was 84 [69, 110] min (Figure 5A). The OT of cluster 1 was significantly shorter than that of cluster 2 (*p* = 0.046). The PDV of cluster 1 was 53 [30, 100] ml, and that of cluster 2 was 80 [40, 130] ml (Figure 5B). The PDV of cluster 1 was significantly lower than that of cluster 2 (*p* = 0.005). Other postoperative variables such as respiratory failure, peptic ulcer postoperation, dysphagia, hoarseness, mental disorder, axial pain, leakage of the cerebrospinal fluid, esophagostoma, wound hematomas, sense of girdle, wound infection, JOA improvement rate >25%, blood transfusion, BV, and the LOS were similar between the clusters (*p* > 0.05) (Supplementary Figures 1 and 2). The operative and postoperative variables are represented by a radargram in Figure 6.

**Table 2:** Postoperative conditions of two clusters of patients.

Postoperative conditions	Overall (*n* = 616)	Cluster 1 (*n* = 469)	Cluster 2 (*n* = 147)	*p*-value
Respiratory failure				0.143
Yes	3 (0.49%)	1 (0.21%)	2 (1.36%)	
No	613 (99.51%)	468 (99.79%)	145 (98.64%)	
Peptic ulcer postoperation				0.633
Yes	6 (0.97%)	4 (0.85%)	2 (1.36%)	
No	610 (99.03%)	465 (99.15%)	145 (98.64%)	
Dysphagia				0.674
Yes	7 (1.14%)	5 (1.06%)	2 (1.36%)	
No	609 (98.86%)	464 (98.94%)	145 (98.64%)	
Pneumonia				0.010
Yes	12 (1.95%)	5 (1.06%)	7 (4.76%)	
No	604 (98.05%)	464 (98.94%)	140 (95.24%)	
Hoarseness				1.000
Yes	4 (0.65%)	3 (0.64%)	1 (0.68%)	
No	612 (99.35%)	466 (99.36%)	146 (99.32%)	
Mental disorder				0.559
Yes	3 (0.49%)	2 (0.43%)	1 (0.68%)	
No	613 (99.51%)	467 (99.57%)	146 (99.32%)	
Axial pain				0.559
Yes	3 (0.49%)	2 (0.43%)	1 (0.68%)	
No	613 (99.51%)	467 (99.57%)	146 (99.32%)	
Leakage of cerebrospinal				0.149
Yes	6 (0.97%)	3 (0.64%)	3 (2.04%)	
No	610 (99.03%)	466 (99.36%)	143 (97.96%)	
Esophagostoma				0.421
Yes	2 (0.32%)	1 (0.21%)	1 (0.68%)	
No	614 (99.68%)	468 (99.79%)	146 (99.32%)	
Wound hematomas				1.000
Yes	6 (0.97%)	5 (1.06%)	1 (0.68%)	
No	610 (99.03%)	464 (98.94%)	146 (99.32%)	
Sense of girdle				0.239
Yes	1 (0.16%)	0 (0%)	1 (0.68%)	
No	615 (99.84%)	469 (100%)	146 (99.32%)	
Wound infection				0.559
Yes	3 (0.49%)	2 (0.43%)	1 (0.68%)	
No	613 (99.51%)	467 (99.57%)	146 (99.32%)	
JOA improvement rate >25%				0.214
Yes	601 (97.56%)	460 (98.08%)	141 (95.92%)	
No	15 (2.44%)	9 (1.92%)	6 (4.08%)	
Blood transfusion				0.280
Yes	31 (5.03%)	21 (4.48%)	10 (6.80%)	
No	585 (94.97%)	448 (95.52%)	137 (93.20%)	

**Table 3:** Postoperative conditions of two clusters of patients.

Postoperative conditions	Cluster 1 (*n* = 469)	Cluster 2 (*n* = 147)	*p*-value
OT			0.046
Medium [P25, P75]	80 [65,100]	84 [69,110]	
BV			0.332
Medium [P25, P75]	100 [50,200]	100 [50,200]	
PDV			0.005
Medium [P25, P75]	53 [30,100]	80 [40,130]	
LOS			0.852
Medium [P25, P75]	8 [6,10]	8 [6,10]	

OT: operation time; BV: bleeding volume; PDV: postoperative drainage volume; LOS: length of hospital stay.

## Discussion

### Orientation and interpretability of unsupervised machine learning

In this study, machine learning algorithms could effectively classify patients with CSM who underwent anterior cervical decompression and fusion into two clusters based on 13 preoperative variables. The results of clustering are presented in Table 1. The results suggested that the UMLA accurately grouped patients according to the severity of their illness. This method was specifically designed to integrate clinical data, which is heterogeneous in an unsupervised manner (9). It can cluster patients with similar characteristics (21). The UMLA clusters patients based on their natural characteristics instead of *a priori* knowledge (22). Supervised machine learning algorithms were used in a study by Kwong et al. (23). In that study, random forest regression models were used to predict waitlist dropout among liver transplant candidates. Such methods provide criteria for categorizing patients, such as whether to drop out of the waitlist of liver transplant candidates. Unlike the above study, we focused on the intrinsic properties of the data, which allowed us to investigate the natural characteristics further and highlight the characteristics that were relevant to the hypothesis concerning medical research. This method provided a more meaningful description and distinction between patient clusters within the cohort (24). For example, cluster 1 and cluster 2 distinguished various attributes of clinical importance, such as age, BMI, and preoperative complications. The clinical attributes of the patients were more severe in cluster 2. The symptoms of CSM and the range of cervical spine lesions were efficiently clustered into the two clusters. Patients in cluster 2 had more severe neurological symptoms, more populations had cervical instability and osteoporosis, and more segments of the cervical spine vertebra needed operation during the surgery. In conclusion, unsupervised machine learning could classify patients into two clusters, according to the severity of the disease and their basic clinical attributes.

### Outcomes of medical therapy based on the classification of unsupervised machine learning algorithms

Based on this classification, we further analyzed 18 operative and postoperative variables of the patients to evaluate the treatment outcomes of the patients in different clusters. Regarding the outcome of surgery, we focused on the postoperative variables axial pain, sense of girdle, and JOA improvement rate >25%. These three variables were similar between the clusters. Although the treatment outcomes between the groups were similar, cluster 2 patients were in a worse condition and had more comorbidities. Thus, the conditions of the patients with severe diseases (cluster 2) might improve considerably after surgical treatment, and the level of improvement of the symptoms might be the same as that of the patients with mild diseases (cluster 1). Recent studies supported the results of our study and demonstrated that patients undergoing anterior cervical decompression and fusion experience significant relief, and patients with more severe symptoms experience greater improvement (25,26). For example, Cole's study divided CSM patients into severe, moderate, and mild groups by severity of myelopathy. After surgical treatment, 30% of patients in the severe group and 58% of patients in the moderate group showed improvement in grip strength, while 9% of patients in the mild group showed improvement in grip strength (5). The results of this study suggested that surgical treatment might be equally effective in severely and mildly ill patients. The results showed the effectiveness of the unsupervised machine learning algorithm in classifying CSM based on their heterogeneous clinical characteristics. Local complications, such as hoarseness, leakage of the cerebrospinal fluid, esophagostoma, wound hematomas, and wound infection, were similar between the clusters. This indicated that not only does anterior cervical decompression and fusion improve symptoms in severely ill patients, but the incidence of surgical complications also does not increase with the severity of the illness (27).

The OT and the postoperation drainage volume were higher in cluster 2. These differences were due to the condition of the patients, such as the surgical segments of the cervical spine, which were reflected in the clustering result. The surgical segments of patients in cluster 2 were larger (*p* < 0.001), and the proportion of patients with long-segment surgery (3 or 4 segments) was 35.47% in cluster 2 and 17.48% in cluster 1. Decompression of the cervical spinal cord or nerve root, reconstruction and stabilization of the cervical spine, and maintenance of the alignment of the spine are the aims of cervical spine surgery (28). Patients in cluster 2 had extensive cervical spinal cord compression. To achieve sufficient decompression, the number of segments requiring surgery increased, and the operative time and the postoperation volume of drainage also increased (Table 3 and Figures 5 and 6). Another reason for the higher postoperation volume of drainage in cluster 2 was that 14.97% of the patients in cluster 2 had osteoporosis. Osteoporosis causes osteopenia in the cervical vertebral body and increases bleeding on the bone graft surface during surgery; thus, patients have more postoperative drainage (29). These differences in the operative variables also verified the accuracy of UMLA in classifying patients with CSM who underwent anterior cervical decompression and fusion.

We did not construct a clinical model or an evaluation system for classifying the patients, as they require external validation and another novel algorithm. Instead, we assessed the ability of the unsupervised machine learning algorithm to cluster these patients and validate the effectiveness of the postoperative variables.

Our study had some limitations. First, the participants were from a single center. Second, this was a retrospective study, and thus, the data might have selection bias. Furthermore, the preferences and the experience of the surgeon might have influenced the results of the study.

## Conclusion

This study showed the effectiveness of unsupervised machine learning as a novel method for classifying patients with CSM who have undergone anterior cervical decompression and fusion. Our results showed that the unsupervised machine learning algorithm could be used to rationally classify a heterogeneous cohort of patients and form a basis for a data-driven platform that can identify the subgroup of patients who might respond to a particular treatment. However, the feasibility and novelty of unsupervised machine learning algorithms and their value in making clinical decisions should be evaluated in prospective controlled trials.

## Data Availability

The original contributions presented in the study are included in the article/**Supplementary Material**, further inquiries can be directed to the corresponding author/s.
