# Supplementary figures and images for: Machine learning-based clustering in cervical spondylotic myelopathy patients to identify heterogeneous clinical characteristics
Source: Front Surg. 2022 Jul 25;9:935656. doi: 10.3389/fsurg.2022.935656 (PMC9357891; doi:10.3389/fsurg.2022.935656)

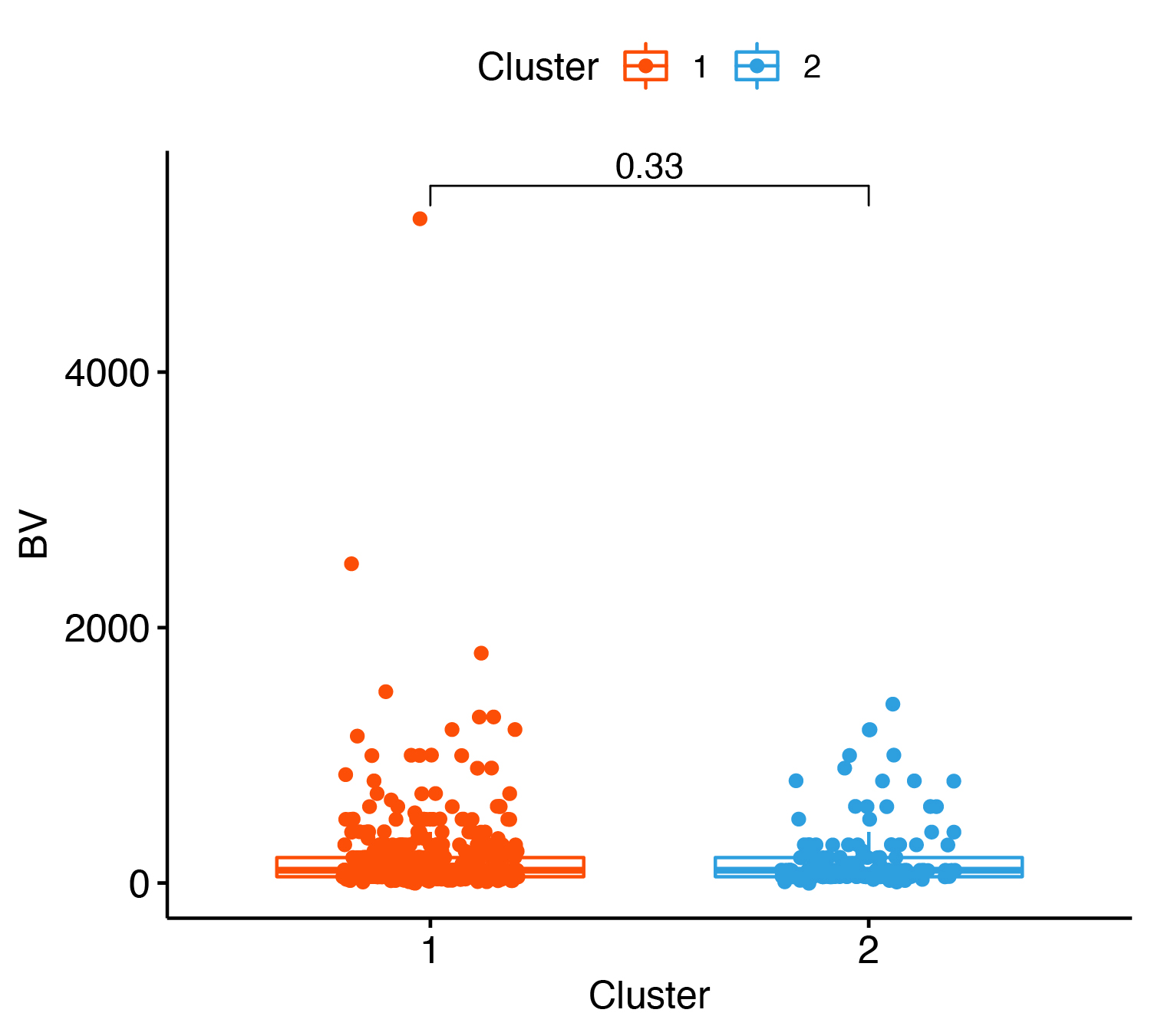

Supplement: Supplementary file 1 [file Image_1_v1.jpeg]

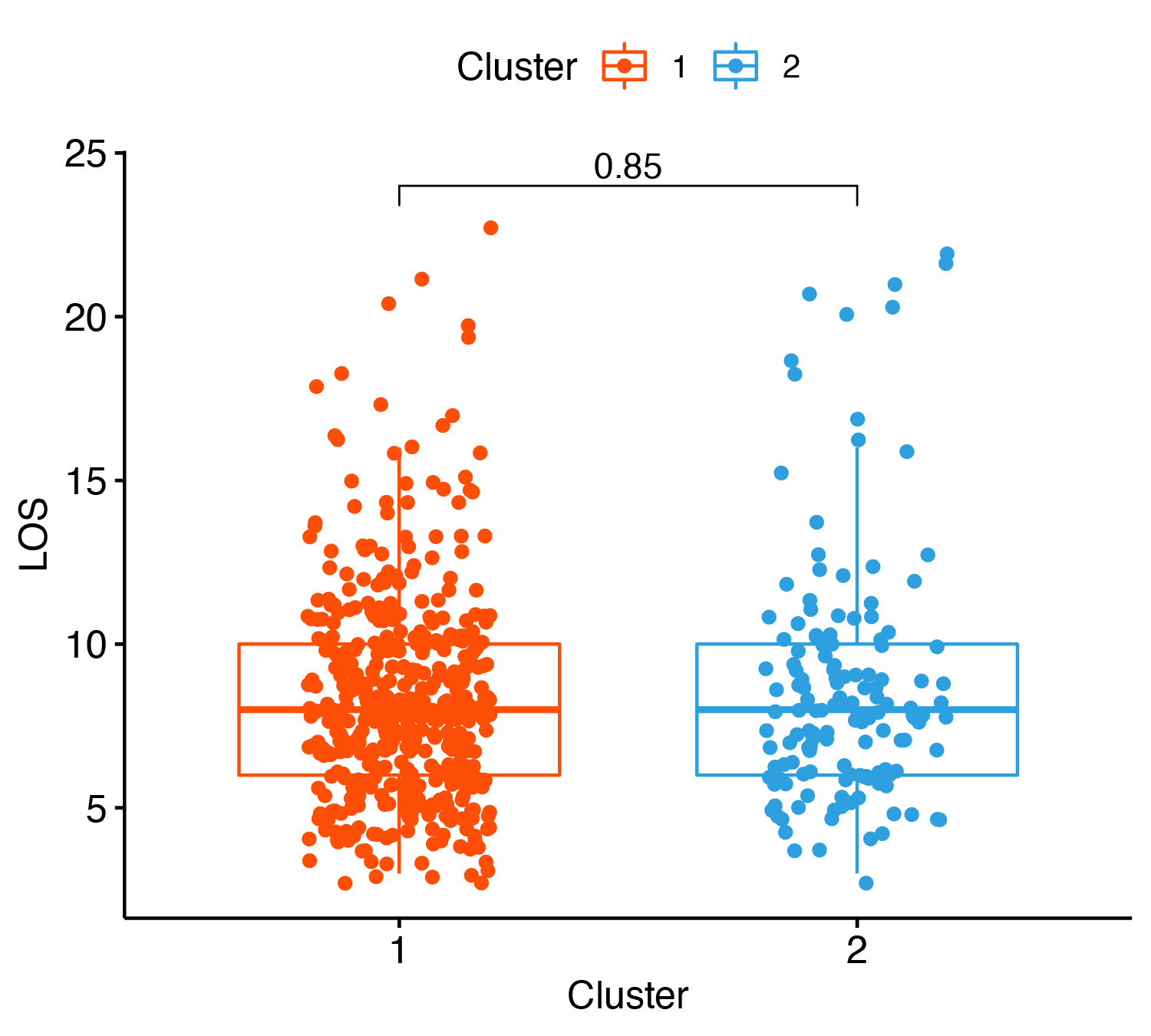

Supplement: Supplementary file 2 [file Image_2_v1.jpeg]
